# Genomic diversity of the locally developed Latvian Darkheaded sheep breed

**DOI:** 10.1016/j.heliyon.2024.e31455

**Published:** 2024-05-16

**Authors:** Dita Gudra, Anda Valdovska, Daina Kairisa, Daiga Galina, Daina Jonkus, Maija Ustinova, Kristine Viksne, Ineta Kalnina, Davids Fridmanis

**Affiliations:** a Latvian Biomedical Research and Study Centre, Riga, LV, 1067, Latvia; b Latvia University of Life Sciences and Technologies, Jelgava, LV, 3001, Latvia

**Keywords:** Whole-genome sequencing, Local breed, Genomic diversity, Latvian Darkheaded sheep

## Abstract

The Latvian Darkheaded is the only locally developed sheep breed. The breed was formed at the beginning of the 20th century by crossing local coarse-wooled sheep with the British Shropshire and Oxfordshire breeds. The breed was later improved by adding Ile-de-France, Texel, German blackheads, and Finnsheep to achieve higher prolificacy and better meat quality. Previous studies have reported the Latvian Darkheaded sheep to be closely related to Estonian and Lithuanian Blackface breeds, according to microsatellite data. To expand our knowledge of the genetic resources of the Latvian Darkheaded breed, we conducted a whole-genome resequencing analysis on 40 native sheep. The investigation showed that local sheep harbor genetic diversity levels similar to those observed among other improved breeds of European origin, including Charollais and Suffolk. Genome-wide nucleotide diversity (π) in Latvian Darkheaded sheep was 3.91 × 10^−3^, whereas the average observed heterozygosity among the 40 animals was 0.267 and 0.438 within the subsample of unrelated individuals. The *N*_*e*_ has rapidly decreased to 200 ten generations ago with a recent drop to *N*_*e*_ 73 four generations ago. However, inbreeding levels based on runs of homozygosity were, on average, low, with F_ROH_ ranging between 0.016 and 0.059. The analysis of the genomic composition of the breed confirmed shared ancestry with sheep of British origin, reflecting the history of the breed. Nevertheless, Latvian Darkheaded sheep were genetically separable. The contemporary Latvian Darkheaded sheep population is genetically diverse with a low inbreeding rate. However, further development of breed management programs is necessary to prevent an increase in inbreeding, loss of genetic diversity, and depletion of breed-specific genetic resources, ensuring the preservation of the native Latvian Darkheaded sheep.

## Highlights

• Latvian Darkheaded sheep are genetically diverse.
• Inbreeding levels among Latvian Darkheaded sheep are low.
• The complex ancestry composition reflects the history of the breed.

## 1 Introduction

As a result of rapidly growing agricultural production, high-yielding animals suitable for intensive farming are predominantly used. Thus, the genetic diversity of animals is decreasing, subsequently depleting genetic resources [[1], [2], [3], [4], [5]]. Furthermore, small ruminant breeding is a significant source of livelihood for many growing rural communities, particularly those living in challenging climatic conditions. This exacerbates the demand for livestock breeds with improved productivity [[6], [7], [8], [9], [10]]. Without careful management of breed improvement, numerous indigenous breeds may be lost [[5], [6], [7],[11], [12], [13]]. Thus, there is a risk of losing genotypes formed in the natural environment that have adapted to local conditions over a long period and are resistant to specific environmental conditions [3,4,14]. Loss of these resources may be detrimental to sustainable agriculture [[3], [4], [5], [6],^10^,^14^]. However, the role of native breeds in local cultural heritage, traditional lifestyle, sense of belonging, and their part in forming local landscapes are no less essential [6,9].

Sheep farming in Latvia has long traditions. According to the records from the 1930s, there were approximately 1.35 million sheep, ranking the country among the largest sheep farmers within the Baltic region [15]. The Latvian Darkheaded sheep (LDS) is a unique breed of local origin developed in Latvia during the early 20th century. The herdbook, issued in 1939, included records on 1016 sheep of the LDS breed [16], and it was created through crossbreeding local coarse-wool sheep with Shropshire and Oxfordshire breeds. For this purpose, 257 Shropshire and 83 Oxfordshire rams were imported from England and Sweden. The primary objective in creating this breed was to produce high-quality semi-fine wool. Over time, efforts to improve the breed led to the introduction of other foreign breeds. In the 1970s, Finnish Landrace (FIN) sheep were introduced to increase prolificacy, whereas in the 1980s, the Ile-de-France and Texel (TEX) meat breeds were introduced to improve the quality of the meat. Furthermore, in the late 1990s, German Blackhead sheep were introduced along with repeated admixture of the Ile-de-France breed [15,17]. The ongoing work to improve the LDS breed continued even at the beginning of the 21st century, when a few animals of Oxford Down sheep were crossbred into the LDS breed [18]. Through careful selection, LDS has been developed to closely resemble the maternal breed, with a medium to large height, sufficient muscular development, strong legs, and hooves; both rams and ewes are polled. Their body wool is white, but the hair on the head and legs is dark brown or black. The breed shows good tolerance for relatively humid local climates. Additionally, ewes of this breed exhibit good fertility, lactation capabilities, and easy lambing, and the lambs are known for their vitality [18].

In the 1990s, because of political changes and the ensuing economic crisis, sheep farming experienced a significant decrease, with total sheep numbers decreasing from 165′000 to 28′000 over the decade. The sheep farming sector has recently recovered, and the number of herds has significantly grown. The contemporary LDS population comprises 22′245 animals belonging to eight major paternal lineages [19]. Currently, the LDS population is not threatened. Still, the geographically limited distribution of the LDS sheep makes the breed vulnerable to changes in local sheep farming practices and breed preferences.

Although it is the only locally developed sheep breed, information on the genetic composition of the LDS is scarce. Previous studies based on microsatellite data have shown close genetic relatedness between the LDS and the two geographically close breeds, Lithuanian Blackface and Estonian Blackhead. The LDS were observed to be the least diverse among the Baltic modern sheep breeds [3,20]. A recent candidate gene study indicated an association between polymorphisms within the myostatin gene locus and muscle mass development [21]. However, genetic variation within the breed at the genome level and the relatedness of the LDS to British and other European breeds have not been investigated. This study was conducted within the framework of a pilot study aimed at the genomic characterization of native ruminant breeds. The major objective of the study was to characterize the genetic diversity and composition of the LDS using whole-genome resequencing data.

## 2 Materials and methods

### 2.1 Animal selection and sampling

In this study, LDS ewes and rams were selected based on pedigree data from the biological farm focused on breeding LDS. The sample group consisted of 40 animals, with 36 ewes and four breeding rams. The affiliation of the included animals with the eight dominant paternal lineages of the LDS breed and their mutual kinship are shown in Supplementary Fig. 1, created using gephi v0.10.0 [22]. Eight sheep samples (four ewes and four rams) were subjected to deep sequencing to reach at least 45× genome coverage, whereas 32 samples (all ewes) underwent shallow sequencing to reach at least 10× genome coverage. The sampling procedure was carried out during routine veterinary examination onsite at the farm where animals were kept. Blood samples of approximately 5 mL from each animal were collected from the jugular vein, usually on the left side of the neck, in ethylenediaminetetraacetic acid (EDTA)-coated vacutainers, and then delivered to the laboratory.

### 2.2 DNA extraction

Upon delivery of the samples to the laboratory, the blood was centrifuged at 4000 rpm for 15 min at 4 °C. Genomic DNA was then extracted from the buffy coat using a previously established routine DNA extraction protocol using the phenol-chloroform method [23]. The quality of extracted DNA was evaluated using 1.2 % agarose gel electrophoresis, whereas the quantity was assessed using the Qubit High Sensitivity DNA assay kit and Qubit 2.0 fluorometer (Thermo Fisher Scientific, USA).

### 2.3 Library construction and sequencing analysis

DNA samples for the whole-genome sequencing analysis were normalized to an initial library input of 500 ng and sheared using a Covaris S220 Focused-ultrasonicator (Covaris, USA) to reach an average fragment size of 400 bp. According to the manufacturer's recommendations, libraries were prepared using the MGIEasy Universal DNA Library Prep Set V1.0 (MGI Tech Co., China). Quality control of the libraries was assessed using the Qubit High Sensitivity dsDNA assay kit on a Qubit 2.0 instrument and the Agilent High Sensitivity DNA kit on an Agilent 2100 Bioanalyzer (Agilent Technologies, USA).

Sequencing depth was calculated to achieve at least 440 million reads per sample for deep sequencing (estimated coverage approximately 45 × ) and 100 million reads per sample for shallow sequencing (estimated coverage approximately 10 × ). Pooled and circularized libraries were used as DNA nanoball (DNB) preparation templates and loaded onto the PE150 flow cell. Libraries were sequenced using the DNBSEQ-G400 sequencer and a DNBSEQ-G400RS High-Throughput Sequencing Set (MGI Tech Co., China) according to the manufacturer's instructions.

### 2.4 Sequencing data processing and variant calling

Quality control and trimming of the obtained paired-end reads were performed using Trimmomatic v0.39 [24] with options LEADING:30, TRAILING:30, and a minimum read length of 36 nt. Quality-filtered reads were then aligned to the *Ovis aries* reference genome assembly ARS-UI_Ramb_v2.0 using the BWA v.0.7.17 MEM algorithm [25]. Mapping results were then converted into the BAM format, sorted by chromosome, and duplicate alignments were removed using SAMtools [26]. Variant calling was performed using GATK HaplotypeCaller [27] and the sheep ARS-UI_Ramb_v2.0 reference genome. All samples were combined into a single genomics database using GATK GenomicsDBImport, and joint genotyping was performed using GATK GenotypeGVCFs. Hard filtering was applied using GATK VariantFiltration to exclude potential false-positive variant calls with the following parameters: QD < 2.0; ReadPosRankSum < −8.0; FS > 60.0; MQ < 50.0; SOR >3.0; MQRankSum < −12.5; QUAL <30; DP > 20. Hard-filtered variants were annotated using the VEP tool [28] and the ARS-UI_Ramb_v2.0 reference. BCFtools was used to calculate the basic statistics of filtered joint sheep genotype files [26]. For comparison with other breeds, whole-genome raw sequencing data from the NCBI Sequence Read Archive (SRA) were obtained for the breeds Texel (TEX; ERR493948, ERR493947, ERR493932), Charollais (CHR; SRR17138945, SRR14949067, SRR14934359), Romanov (ROM; SRR22389467, SRR22389468, SRR22389470), German Mutton Merino (GME; SRR8561010, SRR8561011, SRR8561013), Finnsheep (FIN; SRR19144902, SRR19144929, SRR11657544), Suffolk (SFK; SRR19144775, SRR17165101, SRR14934357), Gotland (GTL; SRR11657690, SRR11657691, SRR11657692), and Dorper (DRP; SRR11657528, SRR11657611, SRR11657612). They were analyzed using the same parameters and references as the LDS breed.

### 2.5 Genomic analysis

The SNP density plot per chromosome of the LDS breed was visualized using package rMVP v.1.0.6 [29]. The degree of polymorphisms in each chromosome, e.g., nucleotide divergence (π), and Tajima's D statistic were estimated using VCFftools v.0.1.17 with a 50 kb sliding window and visualized in each chromosome using the ggplot package within the R environment. Individual heterozygosity was calculated using VCFtools. In contrast, the proportion of heterozygosity was calculated as the ratio of the number of heterozygotic sites divided by the total number of sites. Pairwise comparisons of (i) nucleotide divergence and (ii) heterozygosity between all breeds were conducted using a T-test with the Bonferroni p-value adjustment method with a p-value threshold of 0.05 employing package rstatix v.0.7.2.999, whereas visualization of the results was performed using package ggpubr v.0.5.0.999. For the runs of homozygosity (ROH) analysis, only variants with a minor allele frequency above 0.05 were retained, and ROH were then calculated using PLINK with the following parameters: minimum SNP count of 100, minimum ROH length of 10 kb, and only one heterozygous call was allowed within the ROH. The average ROH in kilobases per individual per chromosome was calculated, and the results of the ROH analysis were visualized using pheatmap v.1.0.12. Package PopLDdecay v.3.42 was used to estimate the linkage disequilibrium degree for all breeds, and the obtained results were visualized using the matplotlib library in the Python environment. Inbreeding coefficients (F_ROH_) were calculated using ROH >100 kb as F_ROH_ = L_ROH_/L_AUTO_, L_ROH_ was the sum of lengths of ROH and L_AUTO_ was the total length of autosomes according to the ARS-UI_Ramb_v2.0 reference genome (2′472′477′635 bp).

### 2.6 Effective population size

To investigate potential historical fluctuations in the LDS population, we applied the GONE software on 40 LDS animals [30]. Before analysis, all indels were removed from the dataset using PLINK. Given that GONE accepts only a maximum of 10 million SNPs per genome and 1 million SNPs per chromosome, we randomly selected a subset of 9 million SNPs using the shell command shuf. This process was repeated 10 times to ensure the possibility of including all SNPs that underwent GONE analysis. During the analysis of effective population size (*N*_*e*_), a maximum of 50′000 SNPs per chromosome and 40 internal replicates run by GONE were set. Genetic distance correction was applied using Haldane's function. Results were visualized using the matplotlib library in the Python environment.

### 2.7 Population structure

Principal component analysis (PCA) and structure analysis were conducted to investigate the genetic background. SNPs in high linkage disequilibrium were removed by PLINK v.1.90b6.21 [31]. The remaining SNPs were used to calculate the principal components, which were visualized using ggplot [32]. Next, the pruned SNP data estimated the individual ancestries using a maximum likelihood method implemented in ADMIXTURE v.1.3 [33]. The default parameters (fold = 5) for cross-validation and the lowest cross-validation error were considered the most probable K values. The results were visualized using the base graphics package within the R environment. To represent the genetic relatedness of animals included in the study, a genetic distance tree based on the UPGMA algorithm with 100 bootstrap replicates was built using package poppr v.2.9.3 [34].

## 3 Results

### 3.1 Sequencing results

**Fig. 1:**
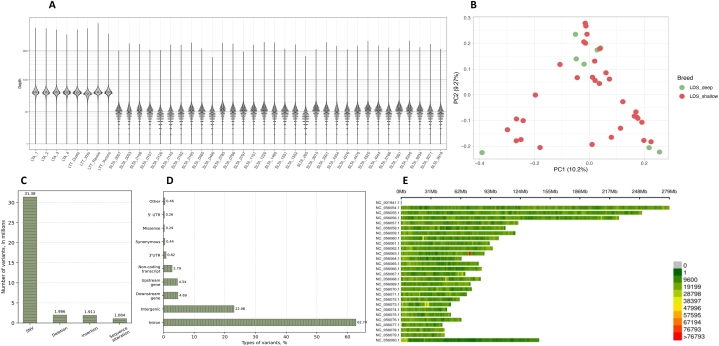
**Sequencing depth of called variants and their distribution.** (A) Sequencing depth of called variants for the LDS sample set. LTA (females) and LTT (males) samples were sequenced with deep target coverage, whereas representatives from SLTA were sequenced with shallow target coverage. (B) Distribution of LDS samples colored by sequencing depth. (C) Distribution of variants in the LDS breed. (D) Consequence types of variants in the LDS breed. (E) The density of SNVs within the 1 Mb window size for each chromosome of LDS breed animals.

The whole-genome sequencing analysis included genomic DNA samples from 40 animals. The concentration of extracted DNA samples ranged between 34.5 ng/μL and 395 ng/μL, with an average of 137.5 ± 88.8 ng/μL (Supplementary Fig. 2). Eight animals representing major LDS lineages were sequenced with high coverage, resulting in a total of 1.8 billion sequences for female LDS samples. On average, each sample yielded 456′410′750 sequences (with a minimum of 448′830′475 and a maximum of 466′026′689), ensuring roughly 52.66× genome coverage (ranging from 51.79 × to 53.77 × ). For four dominant LDS rams, sequencing yield was comparable with a total of 1.8 billion sequences and an average of 445′789′713 sequences per sample (a minimum of 392′364′499 and a maximum of 466′908′888), resulting in a mean genome coverage of 51.44 × (ranging from 45.27 × to 53.87 × ) (Fig. 1A, Supplementary Table 1). The remaining 32 LDS female samples were selected for shallow sequencing, generating a total of 3.5 billion sequences with an average of 109′979′257 per sample (a minimum of 80′323′700 and a maximum of 131′324′165). The genome coverage obtained with shallow sequencing analysis ranged between 9.27 × and 15.15 × , averaging 12.69 × (Fig. 1A, Supplementary Table 1). Samples sequenced at high depth co-segregated with those analyzed using the shallow sequencing approach, thereby supporting the validity of sample pooling for analyzing the breed's genetic composition (Fig. 1B).

After quality filtering, 31′380′115 SNPs and 4′822′511 indels were retained (Fig. 1C). Of a total of 1′665′536 multiallelic sites, 178′195 were identified as multiallelic SNPs. The number of high-quality SNPs called was similar between genomes sequenced with high coverage and those analyzed with the shallow sequencing approach. The mean whole-genome transition-to-transversion ratio (ts/tv) was 2.38. The majority of the called variants, approximately 86 %, were distributed across intergenic regions and introns. Among genetic variants observed in protein-coding parts of the genome, 57.8 % did not affect the amino acid sequence, whereas 38.1 % were classified as missense variants. The SNP distribution across 26 ovine autosomes and the X chromosome and the functional consequences of SNPs are summarized in Fig. 1D and Fig. 1E.

### 3.2 Genomic diversity in the LDS breed

**Fig. 2:**
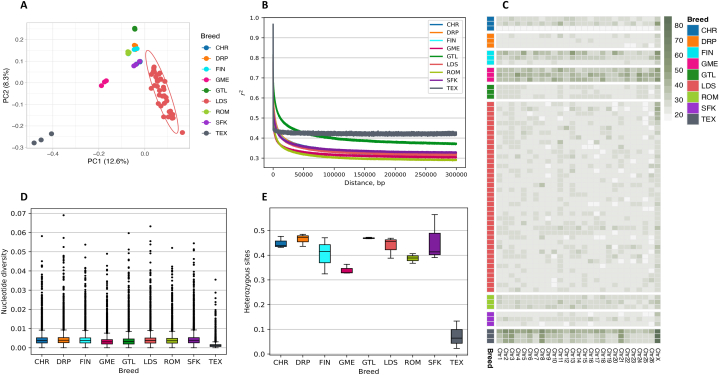
**Genome diversity and linkage disequilibrium (LD) per breed.** (A) PCA analysis of all breeds included in this study. (B) LD decay per breed. The LDS sample set was randomly reduced to contain three samples per breed. (C) Average runs of homozygosity (ROH) per chromosome in each breed. Rows represent samples. Green color represents longer ROH in kilobases gray–shorter. (D) Genome-wide nucleotide diversity for all breeds, where the LDS sample set was randomly reduced to contain three samples. (E) Genome heterozygosity for all breeds, where the LDS sample set was randomly reduced to include three samples. Abbreviations: CHR–Charollais; DRP–Dorper; FIN–Finnsheep; GME–German Mutton Merino; GTL–Gotland; LDS–Latvian Darkheaded sheep; ROM–Romanov; SFK–Suffolk; TEX –Texel sheep.

The genomes of LDS breed animals exhibited moderate genetic variation, as indicated by genome-wide estimates of nucleotide diversity (π) and heterozygosity. The average nucleotide diversity among LDS was relatively high, at 3.91 × 10^−3^ ± 1.96 × 10^−3^. Autosomes, on average, harbored higher diversity compared to chromosome X (4.0 × 10^−3^ ± 1.93 × 10^−3^ and 2.26 × 10^−3^ ± 1.76 × 10^−3^, respectively) (Fig. 2D, Supplementary Fig. 3). However, observed heterozygosity was slightly, though significantly, lower than expected (H_o_ = 0.267 ± 0.017 versus H_e_ = 0.275 ± 6.9 × 10^−6^, paired *t*-test *p* = 0.006). The most genetically diverse individuals had heterozygosity levels up to 0.293, whereas the lowest observed heterozygosity was 0.229 (Supplementary Table 1 and Supplementary Fig. 4). For comparison, genetic variation estimates from randomly selected subsamples representing each breed, including three unrelated animals from LDS, suggested similar genetic diversity to that observed for the widespread CHR breed (π = 3.91 × 10^−3^ and H_o_ = 0.438 versus π = 3.99 × 10^−3^ and H_o_ = 0.449, respectively), followed by FIN (π = 3.97 × 1^−3^, H_o_ = 0.403) and SFK (π = 4.07 × 10^−3^, H_o_ = 0.456). The least diverse was the specialized meat sheep breed TEX (π = 1.23 × 10^−3^, H_o_ = 0.074) (Fig. 2E, Supplementary Figs. 5 and 6).

For the LDS breed, out of 628′315 ROH, 3.29 % were at least 100 kb long. On average, a single LDS genome harbored 517 ± 146.4 ROH longer than 100 kb. The number of ROH per animal ranged from 283 to 954. The two least diverse breeds, TEX and GME sheep, had from 2956 to 4581 and from 1001 to 1457 ROH per animal, respectively. The length of ROH reached 1604.2 kb (mean 192.3 ± 118.1) for TEX and 992.8 kb (157.5 ± 70.2) for GME, whereas for LDS, the maximum estimated ROH length was 980.5 kb (mean 149.7 ± 52.8) (Fig. 1, Fig. 2C).

Despite low heterozygosity, average inbreeding levels among LDS were moderate, with F_ROH_ 0.031 ± 0.009 (median = 0.029, with a minimum of 0.016 and a maximum of 0.059). However, three of the four dominant rams used in breeding programs exhibited inbreeding levels higher than the average, ranging between 0.043 and 0.047, raising potential concerns for the future development of breeding programs (Supplementary Table 1).

For the sheep of the LDS breed, strong linkage disequilibrium (LD) decayed by half its maximum (r^2^ ≈ 0.39) or approximately 18 kb. The rapid decrease was approximately 100 kb, with LD remaining stable at r^2^ ≈ 0.33 (Fig. 2B). Similar LD decay patterns were observed in the improved FIN, CHR, DRP, and SFK breeds. For the SFK breed, LD dropped by half (r^2^ ≈ 0.39) by 17 kb, whereas FIN, CHR, and DRP LD halved by ≈ 20 kb (half the maximum r^2^ ≈ 0.38, r^2^ ≈ 0.39, and r^2^ ≈ 0.38, respectively). The ROM sheep, considered more genetically diverse than other European breeds, displayed a slightly faster LD decay rate, reaching half r^2^ ≈ 0.39 by 6 kb and leveling at approximately 75 kb with r^2^ ≈ 0.30. The two island-born breeds, GTL and TEX, exhibited comparably low LD decay rates. For GTL, LD decayed to half r^2^ ≈ 0.41 at 73 kb reaching plateau of r^2^ ≈ 0.38 at 180 kb. Among notably less genetically diverse TEX animals, r^2^ plateaued at ≈ 0.45 over short distances, suggesting a strong overall LD across the genome (Fig. 2B). However, it is important to note that the random selection of a small number of animals from specific populations represented in genome databases could potentially shift genetic diversity estimates.

**Fig. 3:**
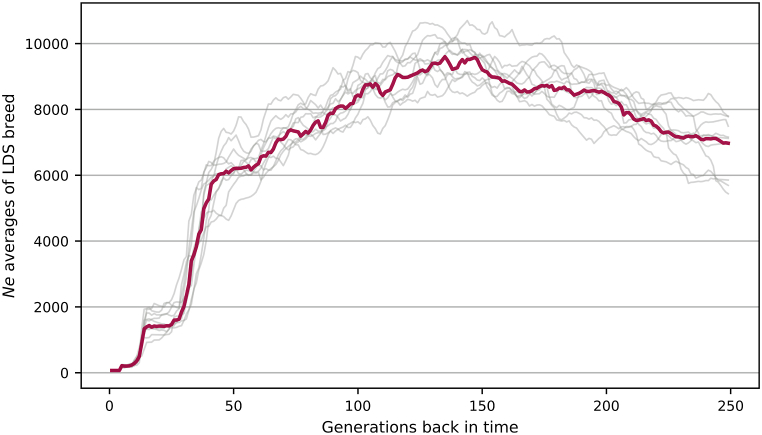
**Effective population sizes for LDS animals up to 250 generations.** Gray lines represent 10 subsets of randomly selected 9 million SNPs, whereas the red line denotes the median measurement of *N*_*e*_ values across all subsets.

The LD-based *N*_*e*_ estimates for the LDS showed a rapid decrease, dropping from 1507 to 229 within the interval between 15th and the ninth generations. The steepest decline of approximately 400 individuals per generation occurred between the 14th and 12th generations (Fig. 3). After the major decline, ten to five generations ago, *N*_*e*_ remained stable above 200. However, the more recent decrease of *N*_*e*_ between the fifth and fourth generations to 73 may be of concern for management of the LDS breed. Assuming a generation time of three to four and a half years, the observed *N*_*e*_ fluctuation patterns correlated with the major demographic changes in the LDS population.

Across autosomes, Tajima's D test produced, on average, moderately positive values (1.195 ± 0.82). Tajima's D values were relatively evenly distributed across autosomes, suggesting a greater influence of demographic processes, such as changes in population size, than selective pressure. The lowest average Tajima's D values were estimated for chromosome 11 (0.961 ± 0.932), and the highest average values were observed for chromosomes 6 (1.334 ± 0.828), 10 (1.314 ± 0.816), and 25 (1.312 ± 0.732). Approximately 8.57 % of the autosomal genome had negative Tajima's D estimates, indicating chromosomal regions potentially containing selective sweeps characteristic of the breed. Correspondingly, with lower nucleotide diversity, the X chromosome displayed a lower genetic variant density than autosomes, and Tajima's D estimates were closer to zero (0.897 ± 1.132) (Supplementary Fig. 7).

### 3.3 Genetic composition of the LDS

Principal Component 1 (PC1) effectively separated the LDS from other European breeds, explaining 12.6 % of the variation. Principal Component 2 (PC2) revealed dispersion among LDS, possibly because of varying proportions of admixture, especially from breeds of British origin. The clusters closest to LDS were those of the British SFK and the French CHR breeds admixed with Leicester sheep. The FIN breed cluster was located further away, whereas more distant breeds included GTL and GME. Contrary to expectations from crossbreeding LDS with TEX, the PCA indicated TEX was the most genetically distant breed (Fig. 2A).

**Fig. 4:**
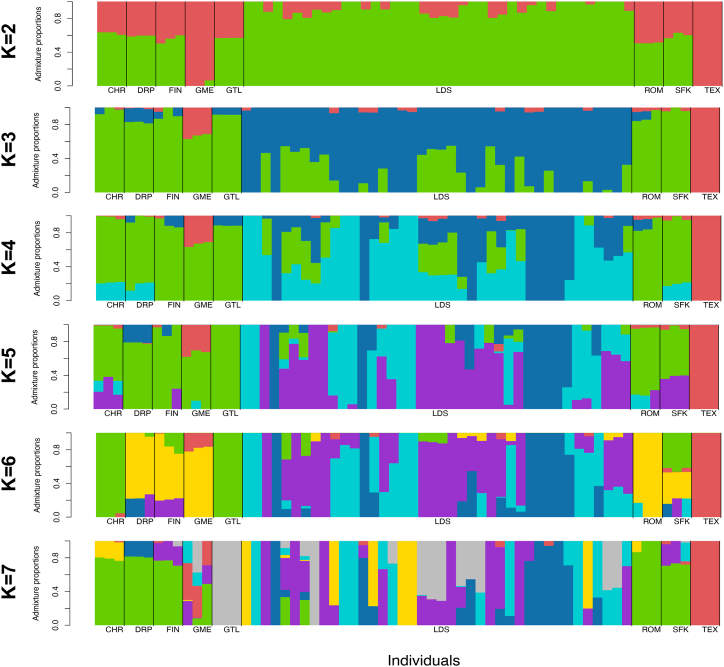
**Historical population structure.** Admixture analysis with K values ranging from 2 to 7. Each animal is represented by a single vertical line divided into K colors. K is the number of clusters assumed, and the colored segment shows the individual's estimated proportion of membership in that cluster. Black lines separate the populations labelled below the figure. Abbreviations: CHR–Charollais; DRP–Dorper; FIN–Finnsheep; GME–German Mutton Merino; GTL–Gotland; LDS–Latvian Darkheaded sheep; ROM–Romanov; SFK–Suffolk; TEX–Texel sheep.

Alongside PCA, admixture analysis was performed to characterize the subpopulation structure within the LDS breed. The optimal number of ancestral fractions, with the lowest error rate, was K = 2 (Fig. 4). Assuming two ancestral components, LDS shared a substantial proportion of ancestry with other breeds of European origin. However, the distribution of ancestral fractions in the LDS breed distinguished local sheep from different breeds. At K values above four, admixture analysis suggested a heterogeneous genetic structure in LDS. The variation in the ancestry composition among animals increased with the growing number of clusters. Certain LDS animals showed traces of TEX ancestry, whereas traces of the FIN breed admixed in LDS were less clear, likely obscured by ancestral fractions shared by the majority of included breeds. Admixture analysis highlighted a genetic component common among LDS animals, distinguishing local sheep from the rest of the breeds.

## 4 Discussion

The LDS is a dual-purpose breed developed for meat and wool production, suited to local climatic conditions characterized by harsh winters. Although not currently under threat, the number of sheep herds is decreasing, and the future of the LDS breed is uncertain. Information on the genetic composition of local sheep populations attributed to the LDS breed is scarce. Initial studies, based on microsatellite markers, indicated that LDS can be distinguished from other Baltic sheep breeds. However, the ancestral background among animals attributed to the breed was highly varied [20]. In this study, we sequenced the genomes of 40 LDS animals to characterize the genetic diversity currently harbored by the local sheep population.

Domestic sheep, in general, have lower genome-wide diversity compared to their wild counterparts such as Asiatic mouflon. According to genome resequencing analysis nucleotide diversity for mouflon samples was approximately a quarter higher than the average for established sheep breeds [1,35]. Estimates of nucleotide diversity at the genome level for LDS were slightly above the range expected for domesticated sheep breeds. A whole-genome sequencing-based study of worldwide sheep populations estimated values ranging between 3.13 × 10^−3^ for the Chinese native Bashibai breed and 1.69 × 10^−3^ for the endangered Svärdsjö sheep, a native Swedish short-tailed breed [35].

Observed heterozygosity among the 40 sheep representing the LDS population (H_o_ = 0.267) was comparatively lower than estimates reported for other native sheep genotyped across Europe. Previously estimated heterozygosity levels for different native breeds from Southern Europe ranged between 0.30 and 0.398. Ho ranged from 0.30 to 0.42 for those from Central Europe, and Ho estimates derived from other studies on sheep breeds originating from northern regions ranged between 0.30 and 0.39 [11,[36], [37], [38], [39], [40], [41]]. For example, the Lithuanian Blackface sheep, considered a modern improved breed with a population of approximately 9000 sheep, has been shown to harbor high genetic diversity, with an average H_o_ of 0.41 [40]. Diversity analyses for most breeds were based on data derived from commercial SNP panels, which tend to produce higher heterozygosity estimates than whole-genome sequencing data because of SNP ascertainment bias [42]. However, heterozygosity levels calculated for three randomly selected unrelated sheep were higher (H_o_ = 0.438), much closer to values observed in other breeds of European ancestry. This could be biased because of the random selection of three highly heterozygous animals. Yet, a more likely explanation is that a relatively high proportion of related animals could have led to underestimating heterozygosity levels within the LDS population [43].

Among the breeds analyzed within the framework of this study, genetic diversity levels retained in the LDS breed were most similar to those of native FIN sheep and the improved CHR breed. Higher genetic diversity was observed for numerous SFK and DRP breeds, whereas GME and ROM breeds were relatively less diverse than LDS. The CHR sheep had the highest diversity estimates, including effective population size and the effective number of founders, among breeds such as SFK, TEX, and the less numerous Galway sheep, according to pedigree records of more than 120′000 purebred animals [44]. Yet another study aimed at pedigree data analysis among German sheep populations listed CHR sheep as less diverse than SFK, indicating that the population of origin might influence conclusions drawn from comparing different breeds [45]. The FIN breed has been reported to harbor moderate levels of genetic diversity, probably because of a comparatively lower population size of approximately 16′000 compared to more mainstream breeds [9]. According to whole-genome resequencing, the FIN population originating from Finland harbored higher nucleotide diversity than GTL and SFK sheep (π 2.79 × 10^−3^ vs. 2.38 × 10^−3^ and 2.47 × 10^−3^, respectively) [35], whereas 50kSNPs chip genotyping data suggested lower diversity among FIN compared to the Australian SFK population (H_o_ 0.371 vs. 0.391) but higher than that found among New Zealand TEX sheep (H_o_ 0.346 vs. 0.330) [39,46]. Heterozygosity levels among FIN were reported to be comparable to those of Lithuanian Blackface sheep (H_o_ 0.39 vs. 0.41) [40]. The two breeds with higher diversity estimates than the LDS sheep, DRP and SFK, are relatively large, internationally distributed breeds. The DRP sheep, as a composite breed, has been previously reported to harbor high genetic diversity [47]. According to in-depth pedigree analysis, internationally widely distributed sheep belonging to the SFK breed are considered genetically diverse [44,45,48]. However, genomic estimates indicate that individual populations may have lost at least a small proportion of the original genetic diversity [35,39]. The relatively less diverse ROM breed, belonging to the group of Northern Short-tailed sheep along with FIN, has been shown to harbor relatively high genome-wide diversity despite displaying the highest coverage with ROH and increased inbreeding compared to other breeds native to Russia [38,49]. However, differences observed in this study among the breeds were non-significant except for TEX. The three reference TEX animals displayed extremely low genetic diversity compared to other breeds. This contradicts reports indicating the genetic richness of the breed, similar to that observed among other internationally distributed breeds such as SFK, with only moderate levels of inbreeding [35,39,44]. Therefore, it is likely that the extreme values derived for TEX were because of random bias in the selection of individual genome sequences from the data pool available online for the TEX breed.

Genome-wide inbreeding levels among LDS were moderate. An average F_ROH_ of 0.031 corresponded to an inbreeding coefficient of 0.030 calculated from pedigree records among lambs born in 2018. These estimates were based on pedigree records for 2295 animals (D. Jonkus-unpublished data), and it indicates that inbreeding levels have remained relatively stable over three years, although there may be certain disparities between the two coefficients associated with the accuracy of pedigree records [50].

Among breeds from neighboring countries, the LDS was listed as the least diverse among other Baltic sheep breeds based on microsatellite allelic richness and heterozygosity levels. However, the inbreeding rate was similar to that of the Lithuanian Blackface [40]. The genomic inbreeding coefficient estimated by SNP-based methods for Lithuanian Blackface was close to zero, indicating that LDS may be more inbred than relatively related improved breeds [3,20,40]. Three out of four resequenced LDS rams had an inbreeding rate above average, likely the consequences of a stronger selection of animals used for breeding, which could become an issue soon if not properly controlled.

For comparison, genome-wide inbreeding levels reported by previous studies in the transboundary sheep breed TEX were approximately between 0.03 and 0.085, depending on the genotyped population [39,51]. Native sheep breeds, shaped by strong artificial and natural selection to strengthen traits like prolificacy, hardiness, and the ability to withstand a harsh climate, like ROM native to Russia and Tibetan Panou sheep, were more inbred than LDS with a respective F_ROH_ of 0.106 and 0.081 [49,52]. Portuguese native sheep breeds were observed to be less inbred compared to LDS. Inbreeding levels among Portuguese breeds ranged from F_ROH_ 0.01 for one of the most abundant local breeds, Bordaleira Serra da Estrela, to F_ROH_ 0.023 in Campanica sheep [36].

The rate of genome-wide LD decay in nine breeds mainly corresponded with patterns expected from other diversity estimates. LD levels among LDS declined with increasing distances at approximately the same rate as in native FIN sheep and improved SFK, CHR, and DRP breeds. The average LD level matched that in FIN but was lower than LD levels in the three improved breeds. The obtained LD decay curves resembled the genome-wide LD decay rate averaged across the genomes of pooled worldwide domestic sheep populations, leveling out above r^2^ 0.3 [35]. Resequencing of domestic sheep showed that maximum LD values halved at 17 kb in improved breeds compared to a 12 kb threshold in landraces. In LDS, LD reached half the maximum at a distance of 18 kb, similar to the mainstream SFK breed, which may indicate that in terms of genetic diversity, LDS could be more comparable to improved breeds than landrace breeds [53].

The numbers of LDS sheep have fluctuated through the decades since the development of the breed, with one of the most notable decreases by approximately 80 % during the 90s, when herds were reduced because of economic changes [19]. The steep drop in sheep numbers appeared to correlate with a drastic 84 % decrease in *N*_*e*_ for the LDS breed. Although these negative changes in *N*_*e*_ could have led to an irretrievable loss of genetic resources characteristic to the LDS breed, between the 10th and fifth generations, *N*_*e*_ remained substantially high in the LDS breed. According to FAO, populations with *N*_*e*_ ≥ 200 could be assumed as not being under threat and harboring enough genetic resources to resist the loss of diversity caused by drift [54]. It would seem that despite variation in numbers through recent decades, the maintained population size, even at its lowest point, has been enough to support substantial genetic variation in the LDS breed, which is often impossible in small purebred populations of local breeds [37,38]. For example, *N*_*e*_ for endangered native breeds, which have been affected by severe demographic changes, like the Czech Wallachian breed, Polish Swiniarka, and Klövsjö and Fjällnäs sheep native to Sweden, ranged between 16 and 32, far below the recommended 50 necessary to prevent detrimental effects of inbreeding [37,38,41]. However, for widespread improved breeds, including SFK, CHR, and TEX, *N*_*e*_ estimates derived from pedigree records and high-density SNP genotyping data were higher than 100 animals per breed, with the lowest *N*_*e*_ for TEX and the highest for CHR exceeding 300 [38,39,44,45]. Among breeds of North European origin, the *N*_*e*_ for FIN sheep 12 generations ago was above the threshold of 200, whereas GTL and ROM had relatively low *N*_*e*_ values of 81 and 61, respectively [38,39].

However, *N*_*e*_ estimates for the LDS showed a further decline in the recent four generations, indicating that current *N*_*e*_ values in the LDS population could be as low as 73 despite relatively high genetic diversity. The progressing loss of genetic resources could reflect a short-term drop in the LDS number to 16′000 occurring twenty years ago [19]. Estimates of *N*_*e*_ above 50 could have been enough to maintain substantial genetic diversity and moderate levels of inbreeding among LDS in the short term [54]. Apart from that, Wang and colleagues found no correlation between *N*_*e*_ and genetic diversity among sheep species [1]. Although not considered critical, a decrease in *N*_*e*_ to 73 indicates an urgent need to adjust the breeding programs to maintain the LDS population [54]. On the other hand, *N*_*e*_ estimates for generations more recent than the fifth generation should be cautiously interpreted. Variation in *N*_*e*_ values may be biased by factors such as population substructure, sampling issues, small sample size, and other factors [55,56]. Therefore, further follow-up studies would be necessary to check that trends in *N*_*e*_ among LDS remain stable.

Although slightly positive Tajima's D values could reflect the population's recovery from a recent drop in sheep numbers, and currently, inbreeding levels are moderate [57], the recommended *N*_*e*_ ranges between 500 and 1000 [54,58]. Breed management strategies should strive to increase *N*_*e*_ to preserve the remaining genetic diversity within the LDS breed in the long term and secure the availability of sufficient resources for further selection efforts.

PCA and admixture analysis successfully separated the genetic composition of LDS from other European breeds into a distinct cluster. However, genetic differentiation was moderate, aligning with the breed's history and origins [[16], [17], [18]]. For an optimal K = 2, LDS sheep showed detectable traces of admixture with sheep of British ancestry, whereas the contribution from FIN sheep was less notable. The introgression of FIN into the LDS breed occurred relatively recently and on a smaller scale to improve the prolificacy of local sheep. During the 1970s, a few FIN rams were imported, and crossbreeding was limited to a few herds. This study may not include direct descendants of the crossbred LDS animals [[16], [17], [18]]. Furthermore, FIN sheep shared ancestry with foreign breeds analyzed here, possibly resulting from the admixture of FIN sheep into other breeds, given the breed's widespread use in improvement efforts [59]. The same holds for crossbreeding with TEX, although the three TEX samples included in the study produced results contradicting those reported elsewhere and may not be typical representatives of the breed.

The LDS breed was formed based on the local coarse-wooled sheep population assumed to be related to the group of Northern Short-tailed sheep [16,18]. Therefore, a larger proportion of shared ancestry between the LDS and two short-tailed sheep breeds, FIN and GTL, could be expected. Nevertheless, the three modern Baltic sheep breeds, including Estonian Blackhead, Lithuanian Blackface, and LDS, were more similar to each other and imported breeds than indigenous old-type sheep belonging to Northern Short-tailed breeds, such as the Estonian native old-type Kihnu sheep [4,20,40,60]. Intensive crossing with mainstream British and German breeds has likely depleted the original genetic background of modern local breeds. Intensive artificial selection could have facilitated further separation of the LDS from ancestral populations [20,61]. This is in line with previous findings based on microsatellite genotyping. The LDS tended to form a tighter, less dispersed cluster compared to the other geographically close breeds, Estonian Blackhead and Lithuanian Blackface, despite similarities in breed origins. The LDS were the least diverse and the most inbred among the three modern Baltic breeds [3,4,20].

For higher admixture coefficients (K > 4), there was a notable increase in ancestry heterogeneity among LDS. This could reflect intentional admixture within the framework of breeding programs and probably a few early uncontrolled crossing events, particularly among smallholder-owned animals [18,20,36]. As shown by PCA, dispersion also favored inter-individual variation among LDS. Furthermore, within-breed variation and random intercrossing between Baltic breeds have been indicated by microsatellite data [20]. However, the study included related individuals, which may have interfered with estimates and artificially boosted the number of ancestral fractions [62]. A larger sample, including unrelated animals, would be necessary before drawing any conclusions on the substructure of the LDS population.

Including related animals is one of the major limitations of the pilot study, potentially causing bias in estimates of genetic diversity. Additionally, the sheep came from a single well-managed herd. Although this may adequately reflect the situation within farms specializing in LDS breeding, where animal mating is strictly controlled following official breeding programs, it omits animals distributed among smallholders. Despite the higher risk of unauthorized crossbreeding, animals kept by smallholders may harbor genetic resources significant to the breed [36]. Furthermore, a few major paternal lineages were underrepresented within the sampled herd. Therefore, more thorough sampling, including different subgroups of animals, would be essential to confirm the current findings and validate generalizability to the entire breed population.

## 5 Conclusions

Whole-genome resequencing analysis has revealed that the locally developed LDS breed harbors substantial genetic diversity, and inbreeding has been maintained at acceptable levels. The genomic composition of the breed reflects demographic processes that have shaped it throughout the decades. LDS exhibit a genetic composition distinct from other contemporary European breeds, although they share high ancestry proportions. Further crossbreeding with mainstream breeds could accelerate the erosion of the LDS-specific signature.

For a comprehensive characterization of the ancestral fractions forming the LDS population, it would be necessary to include other geographically close and potentially historically relevant sheep breeds. All these aspects should be considered when designing management programs to prevent an increase in inbreeding, loss of genetic diversity, and depletion of breed-specific genetic resources. This is crucial for ensuring the preservation of the LDS in the long term, especially given the current locally unstable situation in sheep breeding.

## Ethical statement

The study was conducted following Directive 2020/63/EU on the protection of animals. This study did not need a review by an ethics committee because samples were obtained during routine, non-experimental clinical veterinary procedures on farms. Owners of the animals provided verbal informed consent to participate in the study.

## Consent to publish

All authors approved the manuscript and gave their consent for submission and publication.

## Funding

This study was supported by the 10.13039/501100005375Latvian Council of Science grant No. lzp-2019/1-0075 “Characterization of general and mastitis susceptibility genetic background for Latvia's local (indigenous) breeds of ruminants.”

## Data availability statement

Raw sequencing data have been deposited at the European Nucleotide Archive under study accession No. PRJEB60971.

## CRediT authorship contribution statement

**Dita Gudra:** Writing – review & editing, Writing – original draft, Software, Formal analysis, Data curation. **Anda Valdovska:** Writing – review & editing, Writing – original draft, Supervision, Project administration, Investigation, Funding acquisition, Conceptualization. **Daina Kairisa:** Writing – original draft, Methodology, Investigation, Data curation. **Daiga Galina:** Investigation. **Daina Jonkus:** Methodology. **Maija Ustinova:** Formal analysis. **Kristine Viksne:** Formal analysis. **Ineta Kalnina:** Writing – review & editing, Writing – original draft, Software, Data curation. **Davids Fridmanis:** Writing – review & editing, Writing – original draft, Supervision, Resources, Project administration, Methodology, Funding acquisition, Conceptualization.

## Declaration of competing interest

The authors declare that they have no known competing financial interests or personal relationships that could have appeared to influence the work reported in this paper.
