# Supplementary material for: Genomic diversity of the locally developed Latvian Darkheaded sheep breed
Source: Heliyon. 2024 May 16;10(10):e31455. doi: 10.1016/j.heliyon.2024.e31455 (PMC11130721; doi:10.1016/j.heliyon.2024.e31455)
Supplement: Multimedia component 1 [file mmc1.docx]

**Supplementary Information**

Research article

**Genomic Diversity of the Locally Developed Latvian Darkheaded Sheep Breed**

Dita Gudra^1^, Anda Valdovska^2^, Daina Kairisa^2^, Daiga Galina^2^, Daina Jonkus^2^, Maija Ustinova^1^, Kristine Viksne^1^, Ineta Kalnina^1*^, Davids Fridmanis^1^


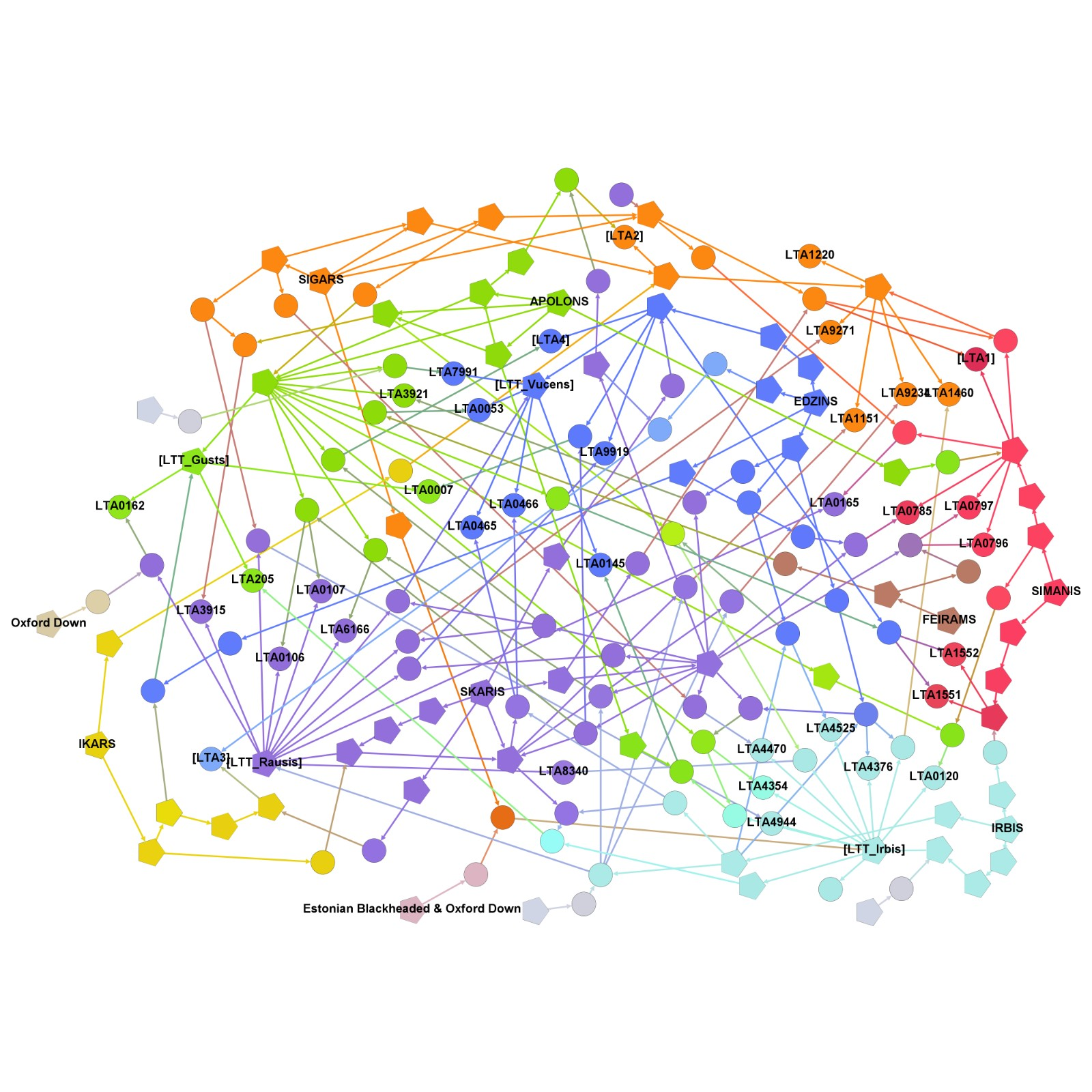


**Supplementary Figure 1**. Graph depicting the affiliation of the included LDS with the eight dominant paternal lineages of the LDS breed and their mutual kinship. Animals analyzed with deep sequencing are marked with brackets. Pentagons represent rams, and circles represent ewes. Colors indicate the degree of kinship between groups of animals with different paternal ancestries. Sheep in light gray have no records of paternal lineages. Each paternal lineage is highlighted in a distinct color. Arrows are drawn from parent to offspring.


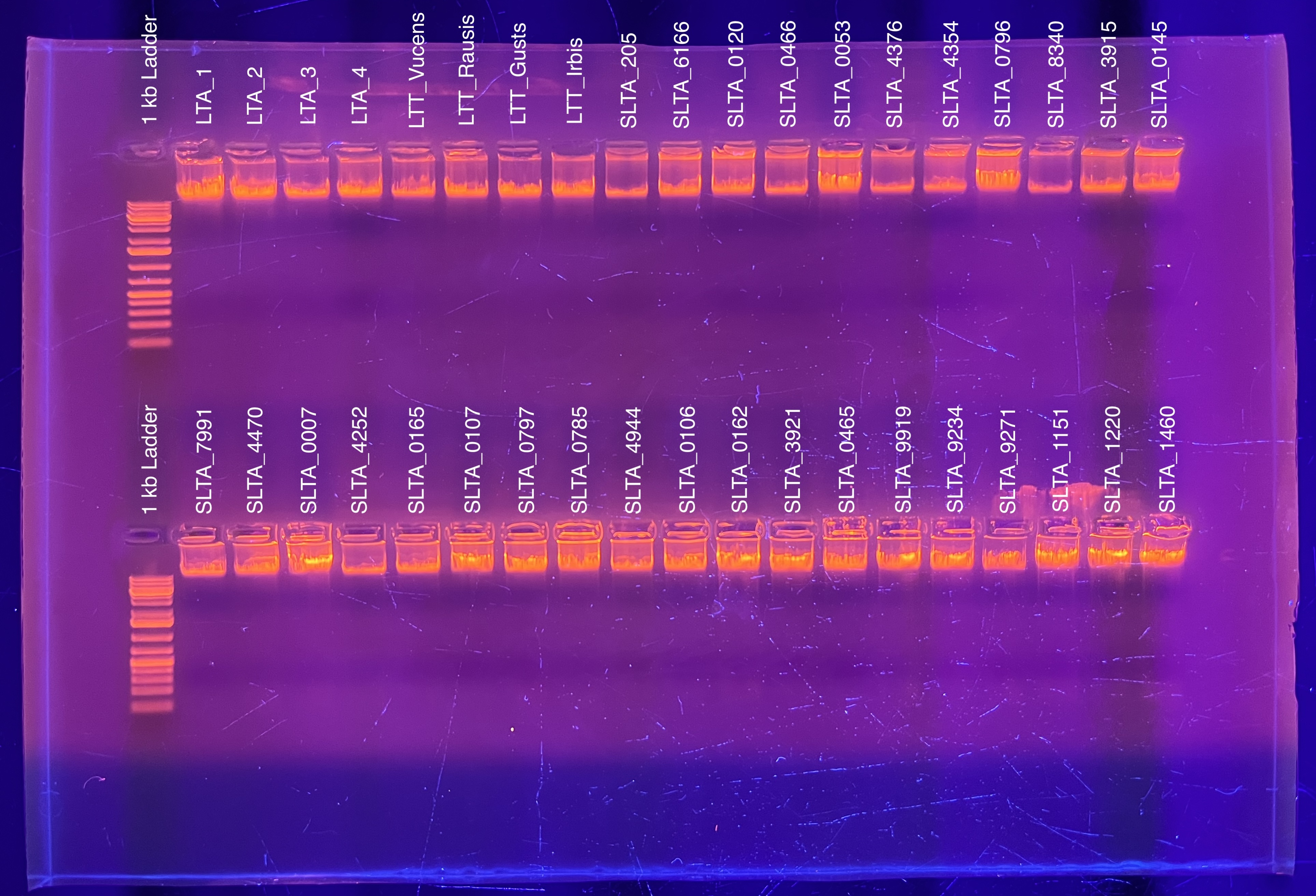


**Supplementary Figure 2.** Agarose gel electrophoresis represents the genomic DNA samples extracted from whole blood using the phenol-chloroform method. Electrophoresis conducted using a 1.2% agarose gel. The first lane of each gel contains the 1 kb Plus DNA ladder (Thermo Fisher Scientific, USA).

**Supplementary Table 1.** Characterization of the sample population

Abbreviations: F_ROH_ – inbreeding coefficients based on runs of homozygosity; CHR–Charollais; DRP–Dorper; FIN–Finnsheep; GME–German Mutton Merino; GTL–Gotland; LDS–Latvian Darkheaded sheep; ROM–Romanov; SFK–Suffolk; TEX–Texel sheep

**
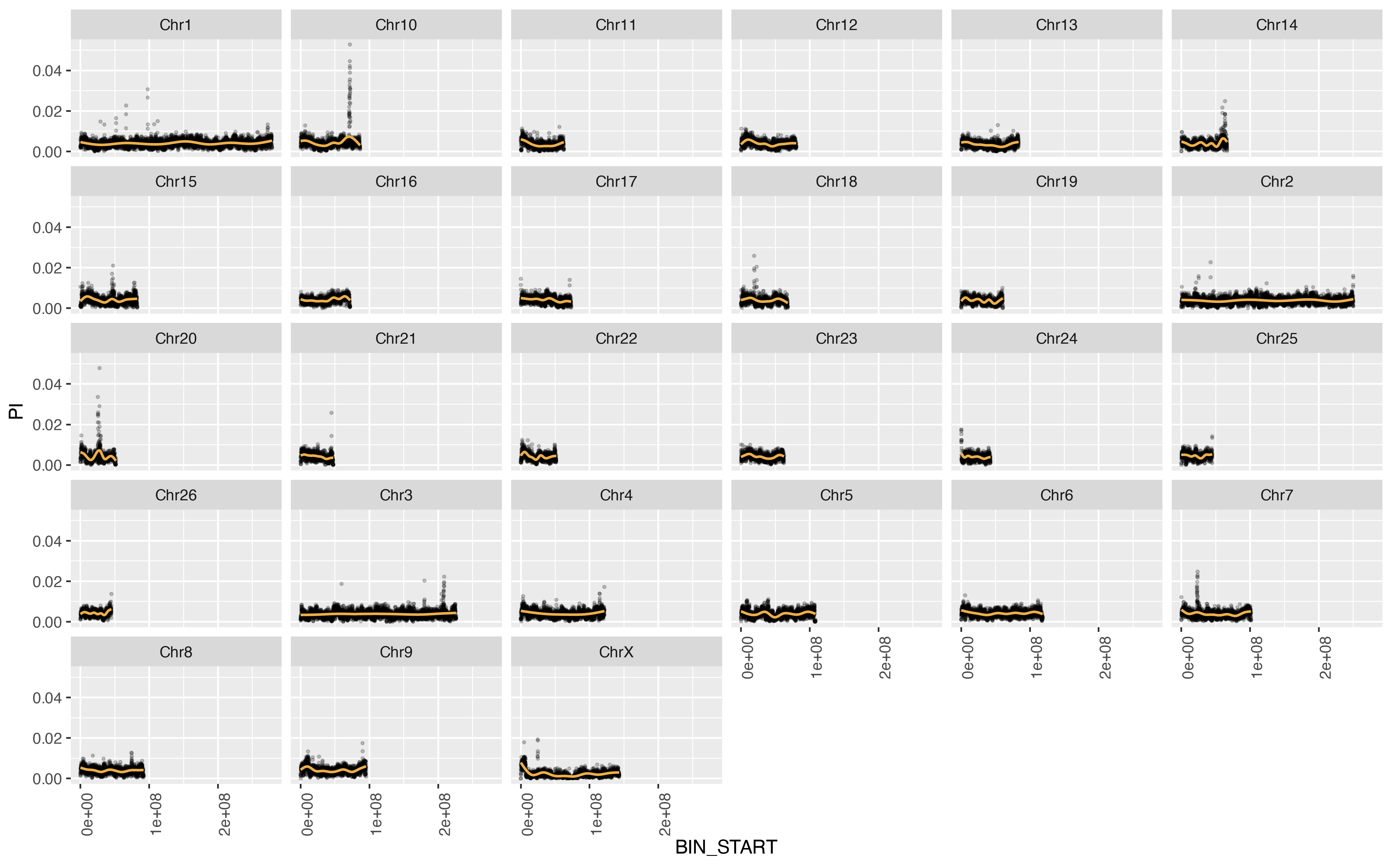
**

**Supplementary Figure 3.** Nucleotide diversity (π) across all chromosomes of the LDS breed (n = 40)


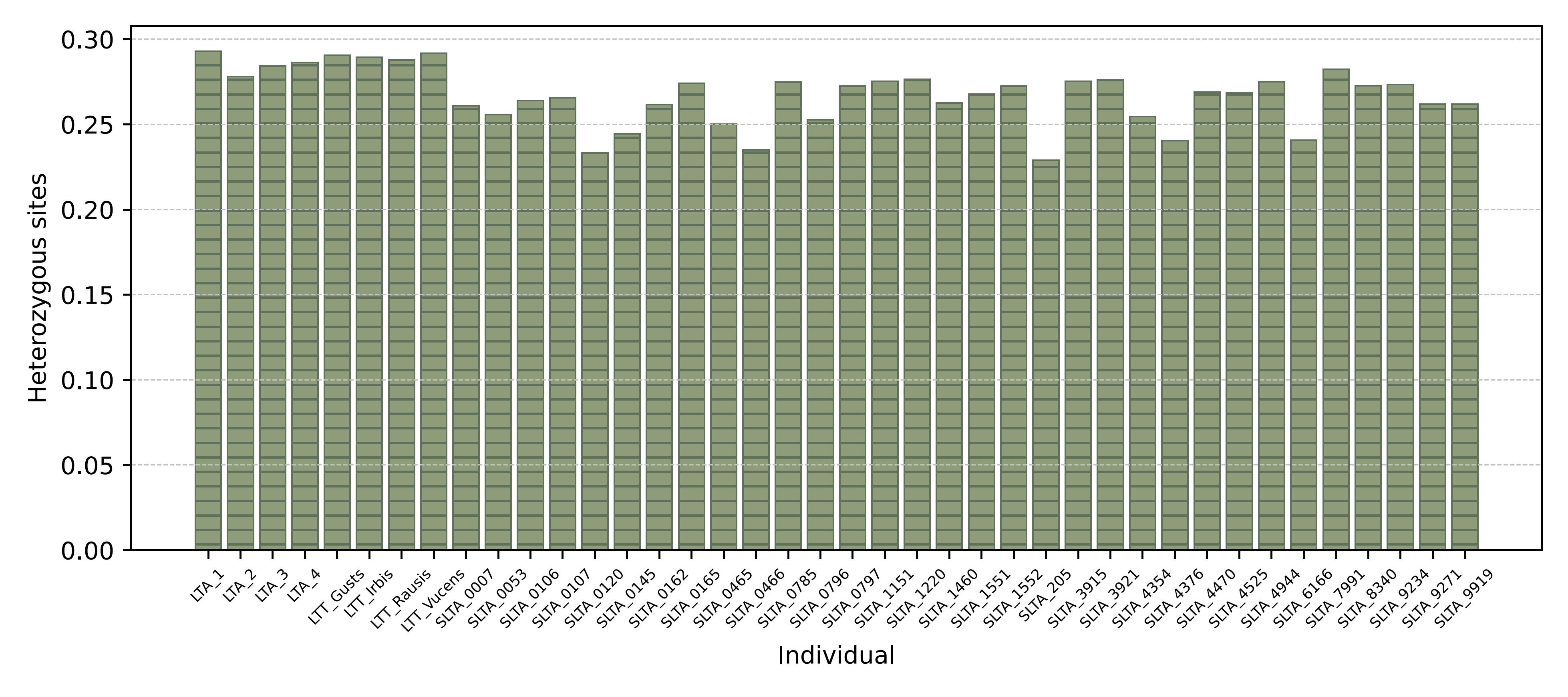


**Supplementary Figure 4.** Genome heterozygosity for all LDS samples included in the current study


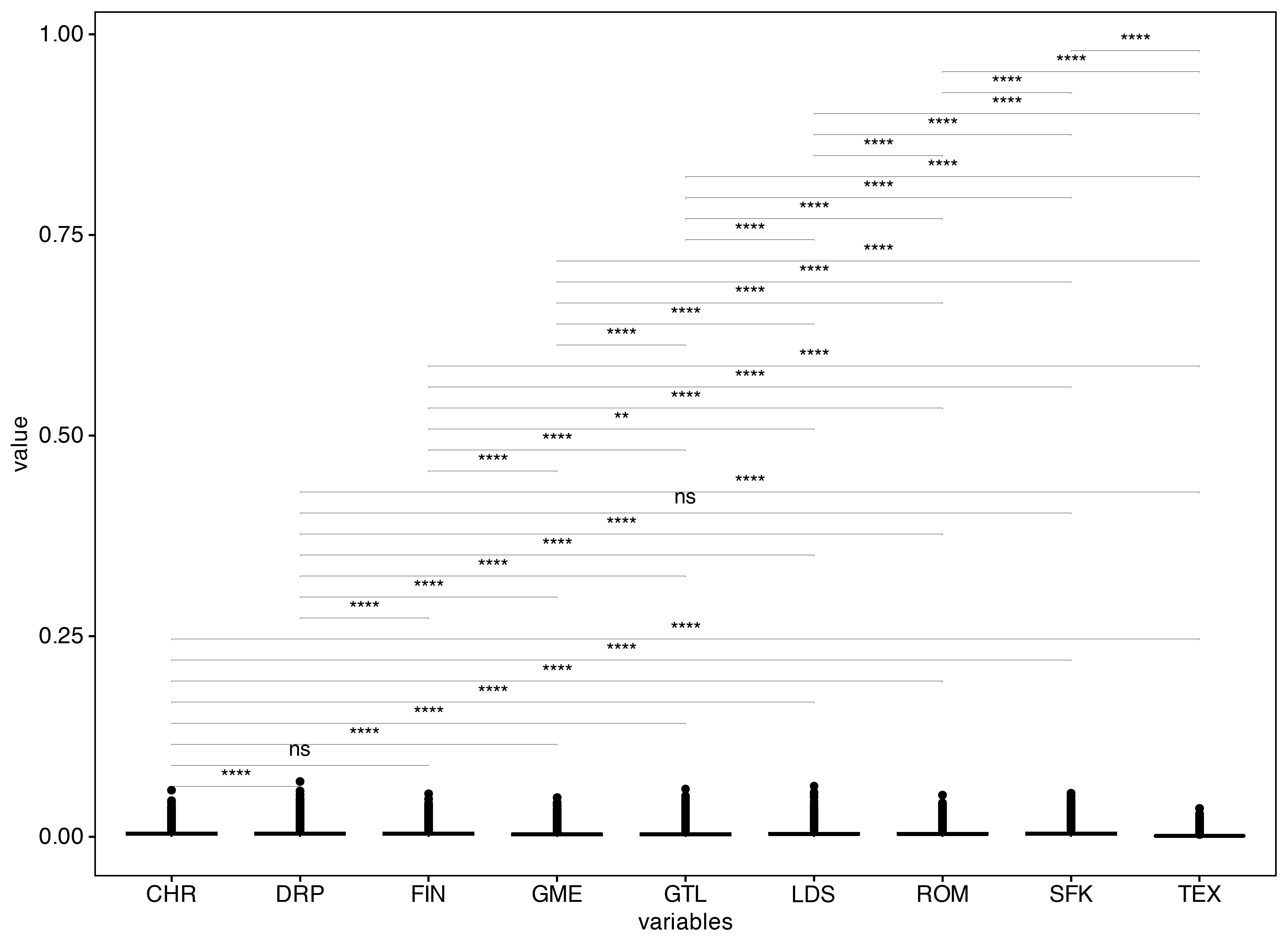


**Supplementary Figure 5.** Genome-wide nucleotide diversity (π) for all breeds. The LDS sample set was randomly reduced to contain three samples. To determine the significant differences between breeds, a pairwise T-test with a Bonferroni p-value adjustment was performed. Significance levels: “ns” – non-significant, “*” – p = (0.01,0.05) “**” – p = (0.001,0.01), “***” – p = (0,0.001), “****” – p <0. Abbreviations: CHR – Charollais; DRP –Dorper; FIN–Finnsheep; GME–German Mutton Merino; GTL–Gotland; LDS–Latvian Darkheaded sheep; ROM–Romanov; SFK–Suffolk; TEX–Texel sheep


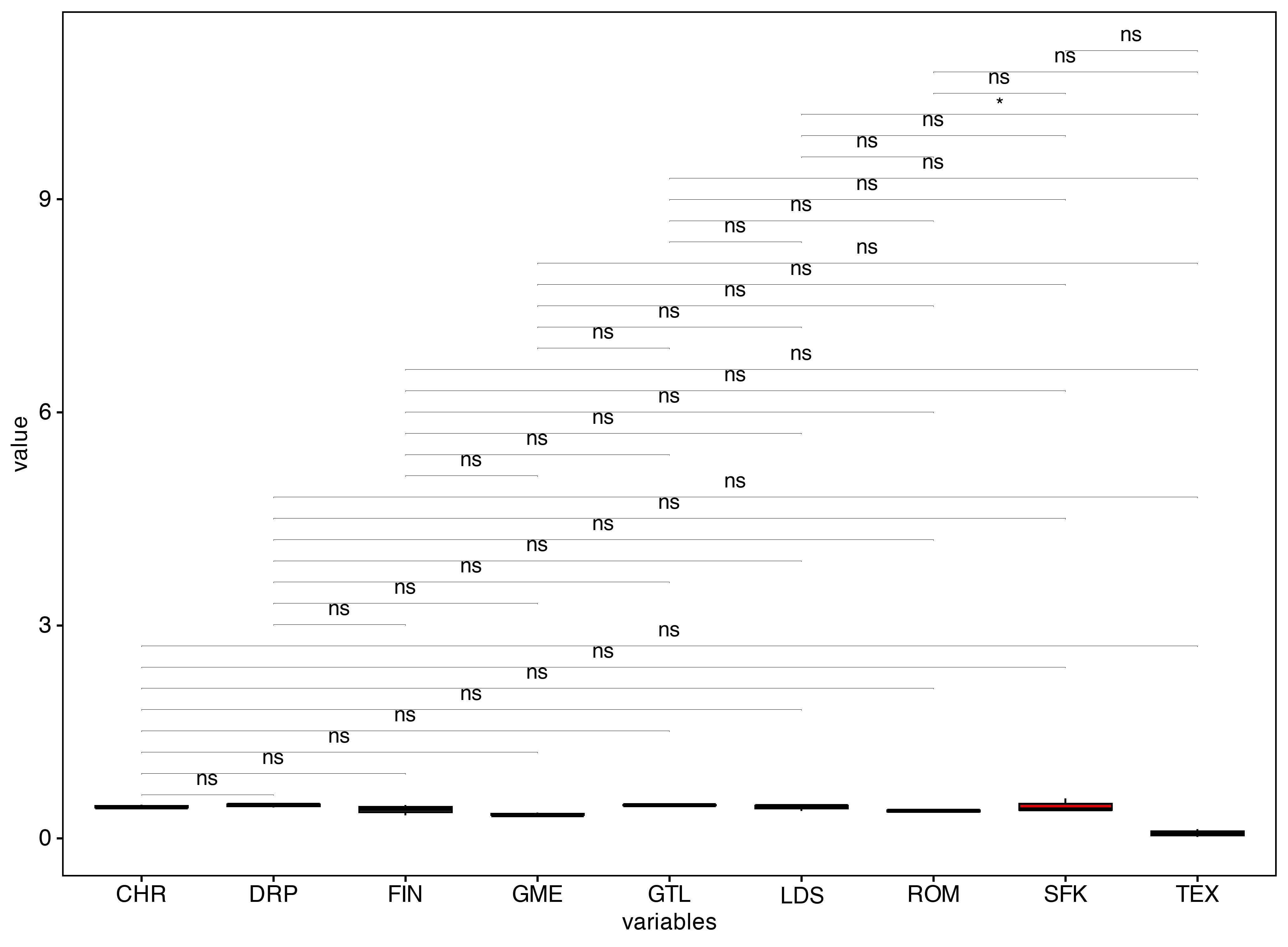


**Supplementary Figure 6.** Genome heterozygosity for all breeds. To determine the significant differences between breeds, a pairwise T-test with a Bonferroni p-value adjustment was performed. Significance levels: “ns” – non-significant, “*” – p = (0.01,0.05). Abbreviations: CHR–Charollais; DRP–Dorper; FIN–Finnsheep; GME–German Mutton Merino; GTL–Gotland; LDS–Latvian Darkheaded sheep; ROM–Romanov; SFK–Suffolk; TEX–Texel sheep.

**
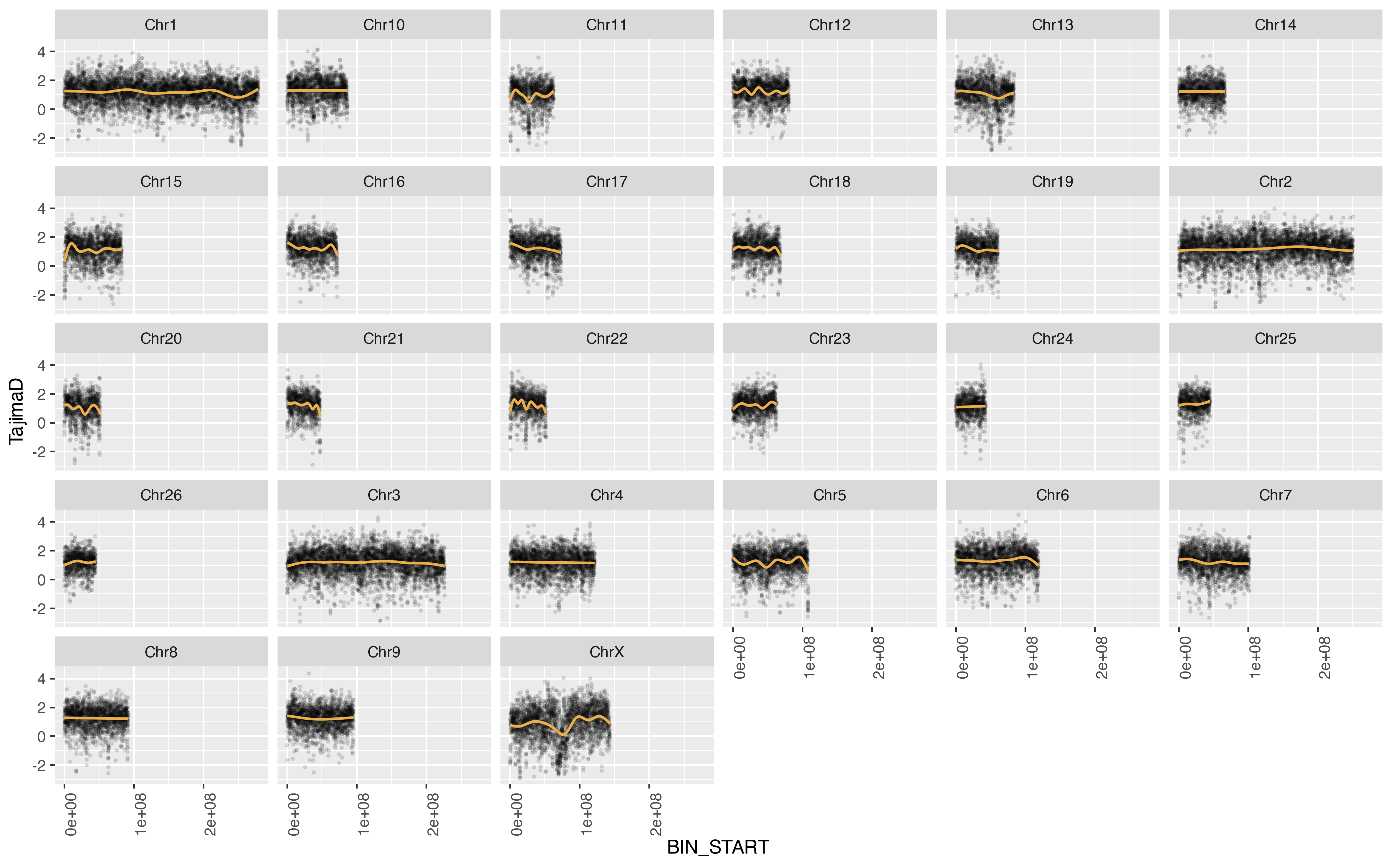
**

**Supplementary Figure 7.** Tajima’s D across all chromosomes of the LDS breed (n = 40)
